# Mint companion plants enhance the attraction of the generalist predator *Nesidiocoris tenuis* according to its experiences of conspecific mint volatiles

**DOI:** 10.1038/s41598-020-58907-6

**Published:** 2020-02-07

**Authors:** Hojun Rim, Sayaka Hattori, Gen-ichiro Arimura

**Affiliations:** Department of Biological Science and Technology, Faculty of Industrial Science and Technology, Tokyo University of Science, Tokyo, 125-8585 Japan

**Keywords:** Behavioural ecology, Plant ecology

## Abstract

Mint plants enable improvement of pest management by attracting herbivore enemies to constitutively released mint volatiles. The generalist predator *Nesidiocoris tenuis* is used worldwide to control agricultural pests, but little is known about whether mint can serve as a companion plant that attracts this predator. To examine this, olfactory responses of *N. tenuis* were assessed using apple mint, candy mint, and spearmint as odor sources. Of the volatiles released by these mint species, candy mint volatiles alone were more attractive than those from undamaged eggplant, and were as attractive as volatiles from eggplant damaged with *Spodoptera litura* larvae. However, no prominent preference for particular volatile(s) among the mint volatiles was shown by *O. strigicollis*. When *N. tenuis* had been previously exposed to candy mint, the predator showed a stronger preference for candy mint volatiles than damaged eggplant volatiles. It was, however, irrelevant whether the predator received benefit or not by predating animal prey during the mint-experience period. In contrast, spearmint-experience increased the preference for spearmint volatiles only when the predator acquired prey during the mint-experience period. These findings suggest that previous exposure of *N. tenuis* to some particular mint species can increase its preference for volatiles from the conspecific mints.

## Introduction

Plants produce and emit volatile organic chemicals (VOCs) in order to resist environment stresses such as heat and light^1^, to deter herbivores and pathogenesis^2–4^, and to attract beneficial organisms, including natural enemies of herbivores^5–7^ and pollinators^8^. The pivotal roles of plant VOCs in the tritrophic system consisting of plant, herbivore, and predator have been intensively researched in a large array of cultivated crops^7^ to be applied for use of biological control of herbivores^9^. Among such applications, **“**companion planting***”*** is one successful example of polyculture in which target plants (TPs) are cultivated with companion plants (CPs) to assist TP growth or protection against pests by attracting beneficial insects or repelling pests^10^. For example, CPs such as mint, basil and marigold work as attractants for herbivore enemies^11,12^. Moreover, an aphid predator, *Cycloneda sanguinea* L. (Coccinellidae), has been shown to be attracted to corianders owing to their pollen and nectar serving as supplementary foods^13^.

Recently, Togashi *et al*. showed that candy mint (*Mentha* x *piperita* L. cv. Candy) and spearmint (*M. spicata* L.) attract *Phytoseiulus persimilis* Athias-Henriot (Phytoseiidae), a specialized predatory mite of Tetranychidae, but not *Neoseiulus californicus* McGregor (Phytoseiidae)^14^, a generalized predator that consumes not only mites but also pollen, thrips, and other tiny arthropods^15^. These results were certainly unexpected because it was initially expected that the generalist *N. californicus* rather than the specialist *P. persimilis* would be responsive to mint VOCs. To the best of our knowledge about companion plants attracting generalist predators, the only other publications are reports showing that basil plants are able to attract the generalist predator green lacewing, *Ceraeochrysa cubana* Hagen (Chrysopidae)^16^, but not another generalist predatory mirid bug, *Macrolophus pygmaeus* Rambur (Miridae)^17^.

In order to further our understanding of generalist predator attraction to CPs, in the current study we assessed the preference of two generalist predators, *Nesidiocoris tenuis* Reuter (Miridae) and *Orius strigicollis* Poppius (Anthocoridae), for mint VOCs using a Y-tube olfactometer assay. These insects are generalists and omnivores which can utilize both diverse animal and plant species as their diets. *N. tenuis* preys on whitefly, thrips, mites, aphids, and eggs/young larvae of lepidopterans^18–22^. *O. strigicollis* is a biocontrol agent for small agricultural pests, including mites, aphids, thrips, eggs or young Lepidopteran larvae^23^. Both predators have been successfully developed for biological control in a suite of agricultural systems^24–28^.

 Based on previous findings by Togashi *et al*.^14^, we focused on candy mint (*M. x piperita* L. cv. Candy), spearmint (*M. spicata* L.), and apple mint (*M. suaveolens* Ehrh.) as CPs, whose VOCs have been categorized into three distinct types, namely, cool-pungent, cool-sweet, and cool-fruity, respectively^29^. In addition, in this study we explored the importance of plastic experiences of exposure to mint odors on predators’ attractivity, because we are aware that olfactory preference of herbivores’ natural enemies can be elevated through associative experiences^30^. The natural enemies come to prefer VOCs released from the herbivore-infested host plant in some cases when they have previously experienced the conspecific herbivore prey on the conspecific host species with the same genotype^31,32^. For instance, it has been shown that *N. tenuis* adults prefer VOCs from eggplants more strongly when they have experienced those VOCs while having their favorite animal prey on eggplant for 4 days^31^. However, *N. tenuis* adults do not show such a VOC preference when they have not had such experience.

Here, we present findings showing the importance of the plastic experience of mint VOCs on the attractivity of *N. tenuis*. Finally, our findings suggest a potential application of mints for pest control that is boosted by the plastic experiences of the carnivore.

## Results

### Attractiveness of mint plant VOCs to *N. tenuis* and *O. strigicollis*

**Figure 1:**
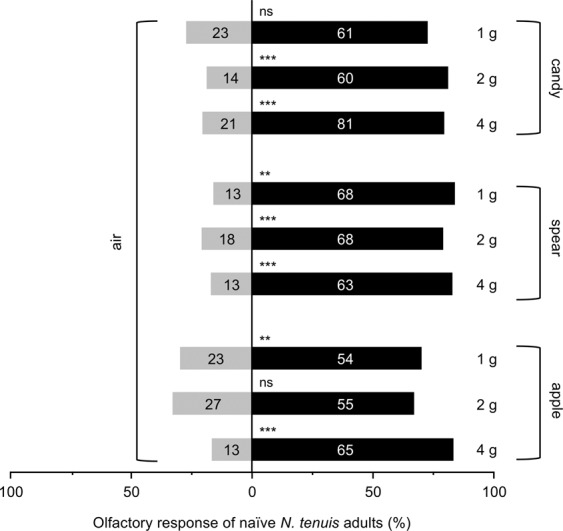
Olfactory response of naïve adults of *Nesidiocoris tenuis* to VOCs from candy mint, spearmint or apple mint plantlets (1, 2 or 4 gram fresh weight) vs. clean air in a Y-tube olfactometer. The numbers within the bars represent the numbers of *N. tenuis* adults that made a choice. Asterisks indicate significant differences based on a generalized linear mixed model (GLMM) with a Wald test (***P < 0.001; **0.001 ≤ *P* < 0.01; ns, *P* ≥ 0.05).

Naïve adults of *N. tenuis* tested in Y-tube olfactometer assays preferred VOCs released from candy mint, spearmint and apple mint (1, 2 or 4 grams fresh weight (gFW)) when compared with clean air (*P* < 0.05, except in the cases of 1 gFW of candy mint [*Z* = 1.934, *P* = 0.053] and 2 gFW of apple mint [*Z* = 1.816, *P* = 0.069]) (Fig. 1). Based on these predator responses, we focused on VOCs from 4 gFW mints for use in subsequent assays.

**Figure 2:**
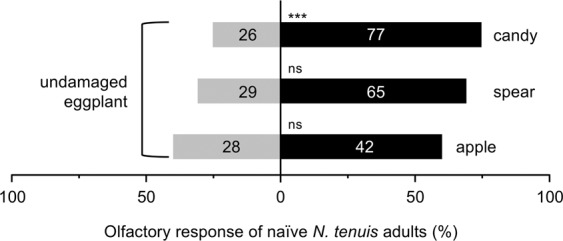
Olfactory response of naïve adults of *Nesidiocoris tenuis* to VOCs from candy mint, spearmint or apple mint plantlets (4 g fresh weight) vs. those from undamaged eggplant in a Y-tube olfactometer. The numbers within the bars represent the numbers of *N. tenuis* adults that made a choice. Asterisks indicate significant differences based on a generalized linear mixed model (GLMM) with a Wald test (***P < 0.001; ns, *P* ≥ 0.05).

*N. tenuis* adults preferred VOCs from 4 gFW of candy mint over those from undamaged eggplant (*Z* = 3.292, *P* < 0.001) (Fig. 2). However, they were not significantly attracted to VOCs from spearmint or apple mint over VOCs from undamaged eggplant (*Z* = 1.497, *P* = 0.134 for spearmint; *Z* = 0.972, *P* = 0.331 for apple mint) (Fig. 2).

**Figure 3:**
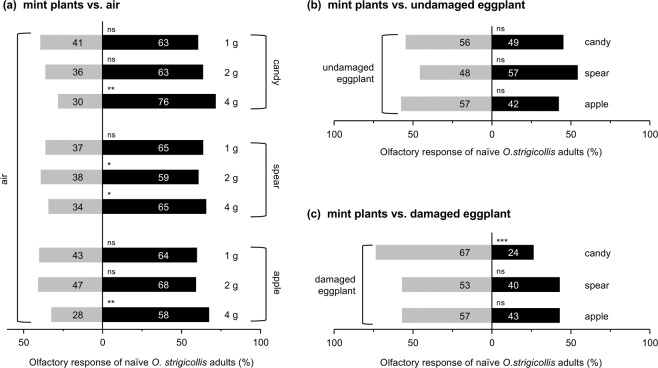
Olfactory response of naïve adults of *Orius strigicollis* to VOCs from candy mint, spearmint or apple mint plantlets (1, 2 or 4 grams fresh weight) vs. clean air (**a**), VOCs from mint plantlets (4 grams fresh weight) vs. those from undamaged eggplants (**b**) or VOCs from mint plantlets (4 grams fresh weight) vs. those from damaged eggplants (**c**) in a Y-tube olfactometer. The numbers within the bars represent the numbers of *O. strigicollis* adults which made a choice. Asterisks indicate significant differences based on a Generalized linear mixed model (GLMM) with a Wald test (***P < 0.001; **0.001 ≤ *P* < 0.01; *0.01 ≤ *P* < 0.05; ns, *P* ≥ 0.05).

Another generalist predator, *Orius strigicollis*, was not attracted to mint VOCs in comparison to undamaged or damaged eggplant VOCs, although it preferred VOCs released from 4 gFW of candy mint, spearmint and apple mint when compared with clean air (*Z* = 2.584, *P* = 0.009 for candy mint; *Z* = 2.214, *P* = 0.027 for spearmint; *Z* = 3.165, *P* = 0.002 for apple mint) (Fig. 3). We therefore did not focus on *O. strigicollis* for the subsequent assays.

### Previous experiences of *N. tenuis* affect its preference for candy mint VOCs

**Figure 4:**
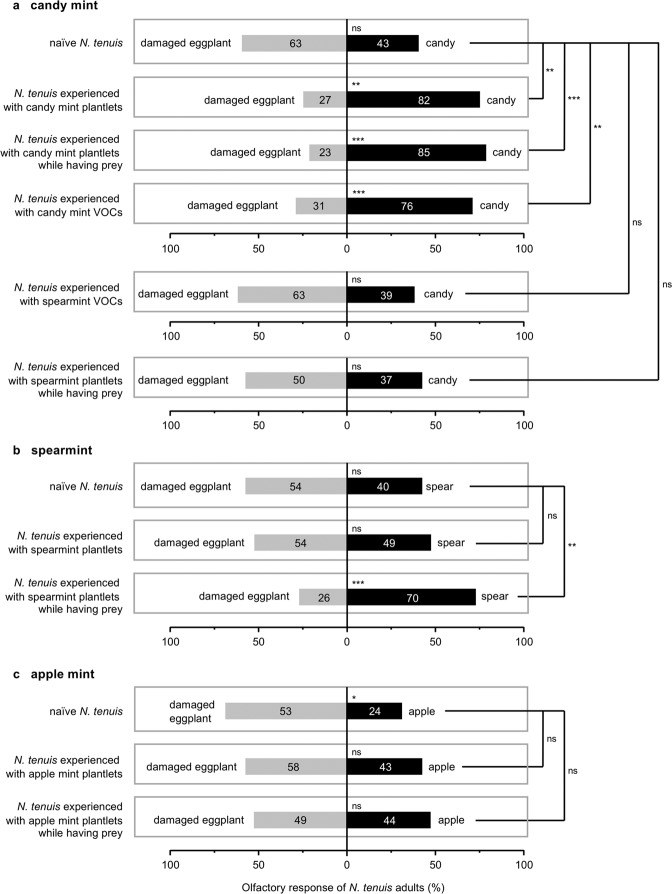
Olfactory response of *Nesidiocoris tenuis* adults to VOCs from candy mint (**a**), spearmint (**b**), or apple mint (**c**) vs. those from eggplant damaged with *Spodoptea litura* larvae for 24 h. We used *N. tenuis* that was naïve to the respective target of mint sp. or *N. tenuis* that had been exposed to the same or different species of mint plantlets or mint VOCs while being provided with animal prey or not. The numbers within the bars represent the numbers of *N. tenuis* adults that made a choice. Asterisks indicate significant differences between left and right columns. The effects of experiences were analyzed based on the generalized linear mixed model (GLMM) with a Wald test (***P < 0.001; **0.001 ≤ *P* < 0.01; *0.01 ≤ *P* < 0.05; ns, *P* ≥ 0.05).

Naïve adults of *N. tenuis* were equally attracted to VOCs from candy mint and to those from eggplant damaged by *S. litura* larvae for 1 day (*Z* = 1.409, *P* = 0.159) (Fig. 4a). The attractivity of candy mint VOCs was increased when *N. tenuis* had been previously exposed to candy mint plantlets for 3 days: in this case, the predator preferred candy mint VOCs over VOCs from the damaged eggplant (*Z* = 3.154, *P* = 0.0016) (Fig. 4a). The same held true for *N. tenuis* that had been exposed to candy mint plantlets while being provided with animal prey (*E. kuehniell*a eggs) (*Z* = 4.684, *P* < 0.001) (Fig. 4a), indicating that exposure to candy mint simultaneously with either beneficial (with-diet) or unbeneficial (without-diet) experiences was able to increase the preference of the predator for mint VOCs (*x*^2^ = 14.953, *df* = 1, *P* < 0.001 and *x*^2^ = 6.836, *df* = 1, *P* = 0.009, respectively).

Next, in order to examine whether the increased preference was due to the experience of perceiving mint VOCs or whether it was due to other factors such as the physical experience of staying on a mint plant irrespective of mint VOCs, we assessed the performance of *N. tenuis* that had been previously exposed to VOCs from candy mint plantlets covered with mesh. This exposure increased the predators’ olfactory preference for candy mint VOCs (*x*^2^ = 7.619, *df* = 1, *P* = 0.006; Z = 4.208, *P* < 0.001) (Fig. 4a), confirming that mint VOCs served as primary factors that confer the significant subsequent preference of the predator for these VOCs.

Finally, to explore the specificity of the VOC composition for this plasticity effect, we assessed the olfactory response of *N. tenuis* after *N. tenuis* had experienced VOCs from spearmint plantlets covered with mesh while having animal prey or not. The VOC blend of candy mint is qualitatively different from that of spearmint^14^. The assays showed that the spearmint VOC-experience affected none of the responses of *N. tenuis* to candy mint VOCs (*x*^2^ = 0.047, *df* = 1, *P* = 0.828; *Z* = 1.305, *P* = 0.192) (Fig. 4a), indicating the specificity of the mint VOC composition on the experience trait. Similarly, when *N. tenuis* had experienced exposure to spearmint plantlets while having prey, it did not show a preference for candy mint VOCs over damaged eggplant VOCs (*x*^2^ = 0.042, *df* = 1, *P* = 0.838; *Z* = 0.960, *P* = 0.337 (Fig. 4a).

### Do mint VOC experiences of *N. tenuis* affect its preferences toward spearmint and apple mint VOCs?

The naïve adults of *N. tenuis* were not attracted to either spearmint or apple mint VOCs in comparison to damaged eggplant VOCs (*Z* = 1.226, *P* = 0.220 and *Z* = 2.018, *P* = 0.044, respectively) (Figs. 4b and 4c). In contrast to the increase of the preference caused by experience with candy mint plantlets or candy mint VOCs (see Fig. 4a), previous exposure to spearmint plantlets did not result in an increased preference for spearmint VOCs (*x*^2^ = 0.358, *df* = 1, *P* = 0.550) (Fig. 4b). However, when the predators were fed animal prey during spearmint-exposure, they started to prefer spearmint VOCs compared to damaged eggplant VOCs (*x*^2^ = 9.229, *df* = 1, *P* = 0.002; *Z* = 3.802, *P* < 0.001) (Fig. 4b). In contrast to the effects of experience with candy mint plantlets, previous exposure to apple mint plantlets did not confer a subsequent preference for apple mint VOCs on *N. tenuis*, regardless of whether *N. tenuis* experienced apple mint-exposure while having or not having animal prey (*Z* = 0.518, *P* = 0.604 and *Z* = 0.643, *P* = 0.520, respectively) (Fig. 4c).

The Y-tube olfactometer assay testing *N. tenuis* that had experienced spearmint plantlets while not having consumed animal prey for 3 days and was then supplied with prey for 4 hours immediately before the start of the assays showed no preference of the predator between damaged eggplant VOCs and spearmint VOCs (Supplemental Fig. 1). Thus, we confirmed that the differences between the olfactory responses of the two groups (experienced with mint while having or not having animal prey during this mint-exposure period) were not likely to have been due to whether or not the *N. tenuis* had been starved.

### Predation activity of *N. tenuis* on the host eggplant in the presence of mint

**Figure 5:**
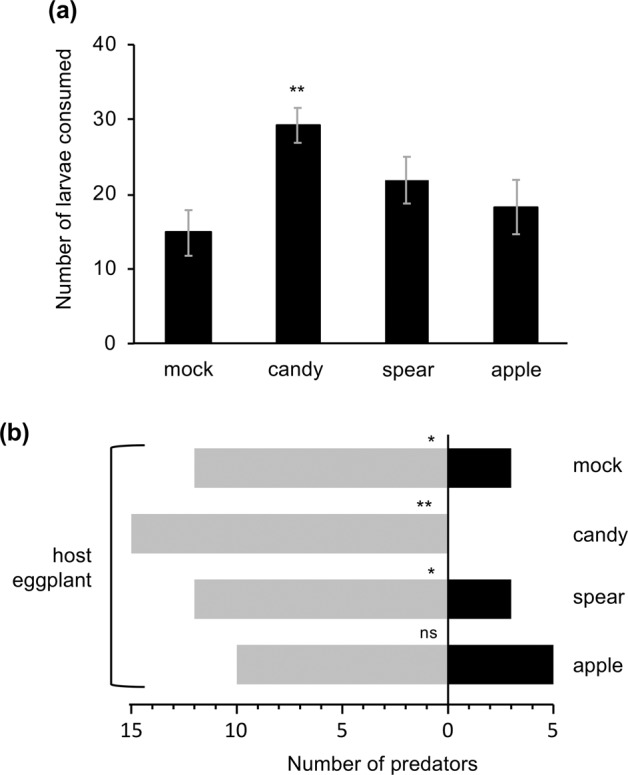
Predation and host-location activity of *Nesidiocoris tenuis*. The number of *Spodoptea litura* larvae consumed (**a**) and localization of predators (**b**) on either a potted host eggplant or each of neighboring candy mint, spearmint, apple mint or mock (undamaged eggplant) plantlets were evaluated 24 h after the beginning of assays. Fifteen replicates were performed. For (**a**), data represent the means and standard errors, and data marked with an asterisk are significantly different from those of mock plantlets, based on one-way ANOVA with post hoc Dunnett’s test (**0.001 ≤ *P* < 0.01). For (**b**), asterisks indicate significant differences based on a generalized linear model (GLM) with a Wald test (***P < 0.001; ns, *P* ≥ 0.05).

Finally, we evaluated the total number of *S. litura* larvae consumed by naïve adult females of *N. tenuis* on the host eggplant in the presence or absence of candy mint, spearmint or apple mint plantlets or mock plantlets (eggplant) for 24 h. *N. tenuis* predated the larvae the most voraciously when candy mint was placed proximately (*P* = 0.005) (Fig. 5a). We also found that significantly more predators observed were located on the host eggplant with *S. litura* larvae, not on candy mint/spearmint or undamaged eggplant (mock) (Fig. 5b). However, when apple mint was placed proximately to the host eggplant, the proportion of the predator population that was located on apple mint was similar to that on located the host eggplant (*P* = 0.206) (Fig. 5b).

## Discussion

Previously, candy mint and spearmint have been shown to act as companion plants for crop target plants in a tri-trophic context, by attracting naïve adults of *P. persimilis*, a predator of *T. urticae*^14^. Of great interest is the fact that this attractivity was comparable to that of VOCs released from *T. urticae*-infested plants.

Here, we showed that naïve adults of the predator *N. tenuis* were attracted to candy mint VOCs with an activity comparable to that of VOCs from *S. litura*-damaged eggplant and higher than that of undamaged eggplant VOCs (Figs. 2 and 4a). We are aware that undamaged eggplants emit very low levels of VOCs consisting of eight major compounds, while damaged eggplants emit much larger quantities and higher quality of VOCs consisting of at least 21 compounds, leading to a stronger attraction of *N. tenuis* and *O. strigicollis* towards VOCs from damaged eggplants over those from undamaged eggplant^33^ (unpublished results about *O. strigicollis* response; Rim *et al*.). The question we then asked is how those predators prefer mint VOCs. This phenomenon is not surprising because there are some reports similarly showing that predators tend to prefer aromatic volatiles released from French marigold, basil, and coriander plants^13,16,34^, although their mechanisms remain to be understood. However, CP-VOCs are not always preferred by predators, as shown here by the lack of any strong preference by *O. strigicollis* (Fig. 3). One of the interesting aspects of predator responses to CP-VOCs is the variability and plasticity shown by predators.

The highlight of the current research was the finding that *N. tenuis* adults showed increased preference for mint VOCs when they had previously experienced the identical mint VOCs (Fig. 4). More importantly, it should be emphasized that the attractiveness of candy mint VOCs to *N. tenuis* adults was elevated when the *N. tenuis* had previously experienced the identical mint VOCs even without having been rewarded by prey or other beneficial supplies (Fig. 4a). To the best of our knowledge, this is the first example of a positive impact of unbeneficial experiences on subsequent responses of a herbivore enemy.

Unbeneficial experiences frequently do not affect or negatively affect a predator’s subsequent performance. For instance, *N. tenuis* adults scarcely preferred VOCs from damaged eggplant if they had not previously experienced the VOCs from eggplant with a reward of prey^31^, which is in agreement with our data for the case of spearmint-experience (Fig. 4b). Moreover, *P. persimilis* and *Anthocoris nemoralis* have been reported to be repelled by herbivore prey-induced plant VOCs after they experienced the identical VOCs without any prey-supply^35,36^. Therefore, we considered the possibility that *N. tenuis* may be rewarded by using candy mint plants as hosts, owing to their nutritional and water resources. However, here we showed that exposure of *N. tenuis* to candy mint VOCs without their being hosted directly on candy mint plants, and without any prey-supply, caused an increase of the preference of *N. tenuis* for candy mint VOCs. This suggests that exposure of *N. tenuis* to the unique blend of candy mint VOCs primes the preference of the predator for the identical mint VOCs, regardless of the fact that mint plants themselves are beneficial for the insect.

Moreover, the plastic learning of the candy mint VOC composition by *N. tenuis* must be accurate, as shown by our finding that previous exposure to spearmint VOCs did not prime an increased preference for candy mint VOCs (Fig. 4a). As described above, candy mint VOCs, consisting mainly of menthone, menthofuran and menthol, are totally different from spearmint VOCs, consisting mainly of carvone^14^. Such qualitatively different VOC compositions are likely the cause of the different abilities to prime the preference of *N. tenuis*. However, it remains to be determined which candy mint VOC(s) are responsible for *N. tenuis*’s learning. Alternatively, the composite blend of candy mint VOCs may be responsible for it.

Notably, candy mint VOCs were also found to stimulate the consumption of prey by *N. tenuis* (Fig. 5a). Although we were initially concerned about the possible trapping of *N. tenuis* on mint plants, which might interfere with hosting of *N. tenuis* on the host eggplant, this did not occur, as shown by the findings with candy mint and spearmint (Fig. 5b). All these results strongly support the possibility that mint CPs act as attractant cues but not hosting cues. In addition, candy mint VOCs work as an appetite-promoting cue as well.

## Conclusions

Competing theories of volatile attraction and volatile masking in particular have been suggested to explain responses in the presence of volatiles from multiple plant sources in tritrophic interactions^37,38^. The findings of this study provide a good framework, but the results presented hint that predator responses are neither as predictable nor rigid as other studies have suggested. This report therefore has value both as a specific test of the value of CPs and also as a more basic exploration of the effects of nearby plants on predator responses to host VOCs.

Moreover, the findings of our study provide novel insights into potential application methods using candy mint and spearmint as CPs for pest management in agriculture and horticulture. These CPs are expected to provide powerful applications when mint VOC-experienced *N. tenuis* predators are simultaneously released in the field. However, it remains to be explored whether mint plants in field or greenhouse settings would increase predation or whether they would mask herbivore location. Nonetheless, given the fact that aromatic plants, including mint (*M. canadensis* and *M. haplocalyx*), basil, and marigold, etc., serving as CPs, have been successfully shown to recruit natural enemy arthropods and reduce herbivores in an array of agricultural fields for pear orchards, tomato farms and tea plantations^12,39–43^, it is highly possible that candy mint and spearmint could be likewise applied for pest control against mint-unexperienced and -experienced *N. tenuis* in field agriculture.

Moreover, given the fact that *O. strigicollis* was not responsive to any of the mint species (Fig. 3), as similarly shown for *N. californicus*, a generalist predatory mite^14^, it should be pointed out that mints do not serve as CPs for all predators. Efficient methods for determining which target plants, seasons, and environmental conditions promote pest management will need to be established to enable practical use of mints as CPs for agriculture and horticulture.

## Methods

### Plants

All of the plants used were incubated in climate-controlled rooms at 24 ± 1 °C with a photoperiod of 16 h (80 µE m^−2^ s^−1^). The light period was from 07:00 to 23:00. Apple mint (*M. suaveolens* Ehrh.), candy mint (*M*. x *piperita* L. cv. Candy), and spearmint (*M. spicata* L.) were obtained from gardening shops, cultivated after insecticide treatment, and propagated by the stem-cutting method. For use as odor sources, the mint plantlet(s) (approximately 1, 2, or 4 grams fresh weight [gFW]) were cut and placed in a glass vial filled with water (35 mL) according to the method described previously^14^. Given the fact that there was no remarkable difference between the VOC profile of the cut plantlets and that of the intact plants of any of the mint species (see Supplemental Fig. 2), we confirmed that mint VOCs focused on in this study served as constitutive VOCs, not mechanical damage-induced VOCs.

The seeds of eggplant (*Solanum melongenas* L. cv. Chikuyo, Solanaceae) were planted in soil in plastic pots (4.5 cm in diameter, 5.5 cm high) and grown for 21–28 days until 4 leaves were fully developed. In order to avoid airborne contamination of eggplants with mint VOCs, all of the eggplants were cultivated at least 5 m away from mint plants when the plants were cultivated in the same room.

### Insects

All of the insects used in the current study were incubated in climate-controlled rooms at 24 ± 1 °C with a photoperiod of 16 h (30 µE m^−2^ s^−1^). *N. tenuis* was obtained from Mitsuki Shimomoto and Kazuhide Nakaishi (Kochi Prefectural Agricultural Technology Research Center, Nangoku, Japan) in 2012. In a lidded plastic container (12 cm × 17 cm × 5 cm high, having 5 mesh windows [1 × 1 cm]), about 20 insects were reared with supplies of *Ephestia kuehniella* Zeller (Lepidoptera: Pyralidae) eggs (Agrisect Inc., Inashiki, Japan) as food and *Sedum rubrotinctum* R.T. Clausen (Crassulaceae) leaves as oviposition substrate and water resource. Twice a week, *N. tenuis* eggs on the *S. rubrotinctum* leaves were transferred to another plastic container that was supplied with fresh *E. kuehniella* eggs and *S. rubrotinctum* leaves. We used *N. tenuis* adults 5–10 days after emergence for the experiments. In order to avoid associative learning of the odor of mint volatiles by *N. tenuis* during rearing, *N. tenuis* was reared in seclusion from mint plants and with constant ventilation of the incubator room.

*Orius strigicollis* was purchased from Sumitomo Chemical Co., Ltd. (Tokyo, Japan) in 2011 and reared using the same method as described above for *N. tenuis*.

*Spodoptera litura* Fabricius (Noctuidae) was transferred to our laboratory in 2014 from a culture reared at Sumika Technoservice Co. Ltd. (Takarazuka, Japan). The larvae were reared on artificial diet (Insecta LF, Nihon Nosan Kogyo Ltd., Tokyo, Japan) in a lidded plastic cup (ø 10 cm × 6 cm, having 2 mesh windows [2 × 2 cm]). When larvae reached the final instar, they were transferred to a larger container (1.6 L) with a mesh-covered lid. Finally, about 30 pupae of *S. litura* were transferred to a lidded plastic cylinder (ø 12 cm × 33 cm) and reared until their adult stage. Meanwhile, their oviposition was allowed on a piece of paper that fully covered the inside surface of the cylinder. The eggs and larvae were used for assays or continuous rearing. First-instar *S. litura* larvae were used for the experiments.

### Preparation of mint-experienced *N. tenuis* adults

To prepare *N. tenuis* that had been exposed to mint plants under various different conditions, 30 adult females and 30 adult males were put in a plastic cage (34 cm × 34 cm × 25.5 cm) where two mint plantlets (4 gFW each) were placed with a sufficient amount of animal prey (about 3500 *E. kuehniella* eggs) or without prey for 3 days. For some experiments, to expose *N. tenuis* to mint VOCs without contact with mint plants, the adults were reared in the plastic cage in which two mint plantlets (4 gFW each) covered with mesh were placed with a piece of wet cotton wool for water supply without animal prey for 3 days.

### Y-tube olfactometer assays

Plantlet(s) of mint plants in a glass vial were placed in a glass container (2 L). A potted eggplant plant (about 4 gFW) that was either undamaged or had been exposed to 10 first-instar *S. litura* larvae for 24 h was also placed in a glass container (2 L). Those plants were used as single-odor sources for Y-tube olfactometer assays. Note that *S. litura* larvae were removed just before the start of assays.

The assays were performed according to the method described previously by Rim *et al*.^33^. Briefly, one *N. tenuis* individual was introduced at the starting point (2 cm from the downwind end of the main tube) in the Y-tube olfactometer (3.5 cm inner diameter, 13 cm long for the main tube, and 13 cm long for each branch tube) using an insect aspirator made for handling of these predators. *O. strigicollis* was introduced on the starting point of a Y-wire set inside the Y-tube using a fine brush. When each predator arrived at the end of either side of the Y-tube, we judged that it made a choice. Predators that did not make a choice within 5 min (“no choice” subjects) were excluded from the statistical analysis. The orientation of the odor-source containers relative to the olfactometer arms was changed after every five bioassays. Assays using both 20 males and 20 females were separately carried out as a single replicate in a day. Three replicates using independent plants were carried out on different days (i.e., 120 predators in all). The experiments were performed in a climate-controlled room (24 ± 1 °C).

### Predation assay

Fifty larvae of *S. litura* (within the first day after hatching) were released onto a potted host eggplant (4 gFW) in a plastic container (34 cm × 34 cm × 25.5 cm, with two small mesh windows [12 × 18 cm] and one large mesh window [34 × 25.5 cm]). Either a mint plantlet (4 gFW) or a mock plantlet (undamaged eggplant [4 gFW]) serving as control was then placed in the container 15 cm apart from the host eggplant. A single adult female *N. tenuis* was immediately introduced to the central point between the host eggplant and the mint/mock plantlet, and the number of *S. litura* larvae that survived was counted after 24 h. We also observed whether the predator was present on either the host eggplant or mint/mock plantlet. Before assays, all the females were reared with a leaf of *S*. *rubrotinctum* alone for 48 h. The assays were performed in a climate-controlled room (24 ± 1 °C) with a photoperiod of 16 h (80 μE m^−2^ s^−1^). Fifteen replicates for each set of assays were performed.

### Headspace volatile analysis

VOCs from the potted mint plants and plantlets in a glass vial (4–5 gFW) were collected in a glass container (2 L) using Tenax 60/80 (Gerstel GmbH & Co. KG, Mülheim an der Ruhr, Germany) for 4 h and analyzed by gas chromatography-mass spectrometry (GC-MS), according to the method described previously by Togashi *et al*.^14^.

### Statistical analysis

All of the results of Y-tube bioassays related to response of predators were analyzed with a generalized linear mixed model (GLMM) with a binomial distribution and logit link using the lme4 package^44^ in R version 3.4.2^45^. The independent trails and sex of predators were included in the model as random effects. The effects of experiences were analyzed by comparing the model “without experience” to the model “with experience” using a Wald test.

One-way ANOVA with Dunnett’s test was used for the results of predation assays using multcomp package^46^. A *P* value < 0.05 was considered statistically significant.

## Supplementary information

Supplemental information
