# Supplementary material for: Mint companion plants enhance the attraction of the generalist predator Nesidiocoris tenuis according to its experiences of conspecific mint volatiles
Source: Sci Rep. 2020 Feb 7;10:2078. doi: 10.1038/s41598-020-58907-6 (PMC7005881; doi:10.1038/s41598-020-58907-6)
Supplement: Supplementary file 1 — Supplemental information [file 41598_2020_58907_MOESM1_ESM.pdf]

Mint companion plants enhance the attraction of the generalist predator *Nesidiocoris tenuis* according to its experiences of conspecific mint volatiles

Hojun Rim, Sayaka Hattori, Gen-ichiro Arimura

Department of Biological Science and Technology, Faculty of Industrial Science and Technology, Tokyo University of Science, Tokyo 125-8585, Japan

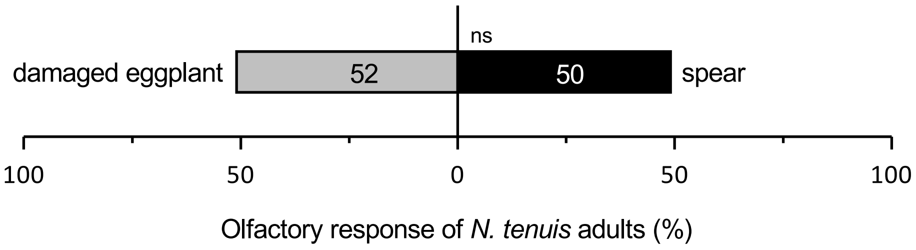

Supplemental
Figure 1

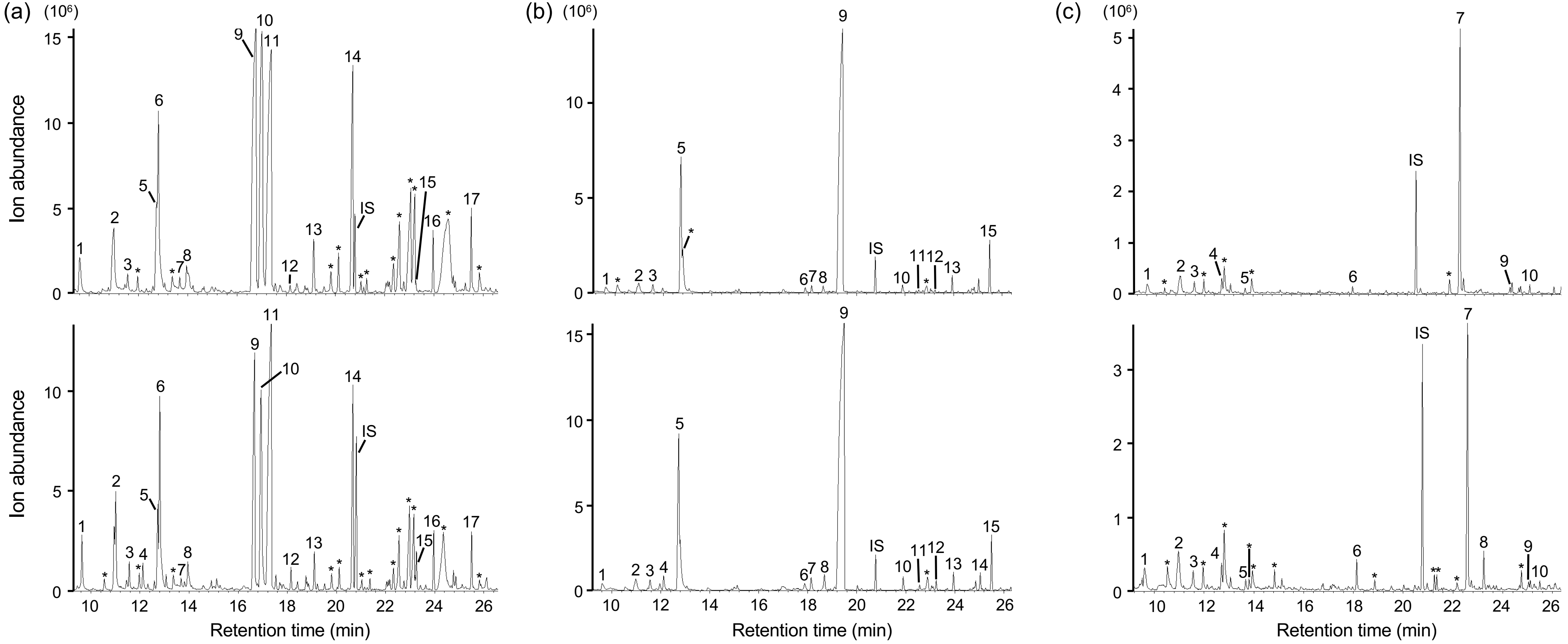

(a)

1. α-pinene
2. β-pinene
3. β-myrcene
4. (*Z*)-3-hexenyl acetate
5. limonene
6. cineole
7. γ-terpinene
8. (*E*)-sabinene hydrate
9. menthone
10. menthofuran
11. menthol
12. decanal
13. pulegone
14. camphene
15. β-elemene
16. (*E*)-β-caryophyllene
17. germacrene D

(b)

1. α-pinene
2. β-pinene
3. β-myrcene
4. (*Z*)-3-hexenyl acetate
5. limonene
6. (*Z*)-dihydrocarvone
7. decanal
8. trans-carveol
9. carvone
10. piperitenone
11. piperitenone oxide
12. β-elemene
13. (*E*)-β-caryophyllene
14. epi-bicyclo-sesquiphellandrene
10. germacrene D

(c)

1. α-pinene
2. β-pinene
3. β-myrcene
4. limonene
5. γ-terpinene
6. decanal
7. piperitenone oxide
8. β-elemene
9. epi-bicyclosesquiphellandrene
10. germacrene D

IS internal standard
* air contamination

Supplemental Figure 2

Supplemental Figure 1. Olfactory response of prey-supplied *Nesidiocoris tenuis* adults to VOCs from spearmint vs. those from eggplant damaged with *Spodoptea litura* larvae for 24 h. *N. tenuis* was exposed to spearmint plantlets while not being provided with animal prey for 3 days. The predators were then supplied with the prey for 4 h immediately before the start of the olfactory assays. The numbers within the bars represent the numbers of *N. tenuis* adults that made a choice. n.s. indicates insignificant differences based on a generalized linear mixed model (GLMM) with a Wald test ($P \geq 0.05$).

Supplemental Figure 2. Headspace VOC profiles of the potted, intact plants and detached plantlets of candy mint (a), spearmint (b), and apple mint (c).
